# Investigations on Beekeeping and Breeding of *Apis cerana* in China

**DOI:** 10.3390/life15010009

**Published:** 2024-12-25

**Authors:** Xinying Qu, Xinru Zhang, Guiqian Zhang, Hanrong Qin, Huixia Zhang, Huiyu Tian, Xiao Chen

**Affiliations:** 1 State Key Laboratory of Resource Insects, Institute of Apicultural Research, Chinese Academy of Agricultural Sciences, Beijing 100193, China; 82101235489@caas.cn; 2 College of Bioscience and Resource Environment, Beijing University of Agriculture, Beijing 102206, China; 202330112016@bua.edu.cn; 3 Gansu Institute of Apicultural Research, Tianshui 741022, China; hczyczgq@163.com; 4 Guangxi Beekeeping Guidance Station, Nanning 530012, China; chsqhr@163.com; 5 Guangdong Agricultural Technology Extension Center, Guangzhou 510520, China; zhxgd2002@sina.com; 6 Yulin City of Animal Husbandry and Veterinary Center, Yulin 719000, China

**Keywords:** *Apis cerana*, traits, biological characteristics, colony management

## Abstract

The worldwide value of the honey bee as an agricultural animal is increasingly being recognized. Not only does the honey bee directly produce useful agricultural products, but also large portions of crops are dependent on the pollination activities of honey bees. *Apis cerana* (*A. cerana*), the native honey bee of China, is widely distributed in the country. Studying the biological environment and colony management of *A. cerana* is important for its conservation and breeding. This study investigated the apiculture of *A. cerana* among a total of 201 beekeepers in the eastern, southern, northwestern and central regions of China and conducted data analysis on the surveyed data. The results showed that the most favorite traits for beekeepers are colony size, colony health and honey production. Compared with *Apis mellifera ligustica*, *A. cerana* is more adaptable to low temperature and scattered nectar sources. The results help to optimize the breeding programs of *A. cerana* and further contribute to substantive breeding accomplishments with honey bees which have enhanced their role in modern agriculture.

## 1. Introduction

The worldwide value of the honey bee as an agricultural animal is increasingly being recognized. The honey bee is not only a direct producer of valuable products [1] but also plays an essential part in pollinating a substantial amount of crops [2]. Approximately 75% of the world’s primary crop species rely on pollinators for the production of fruits and seeds [3]. Furthermore, pollinators play a pivotal role in providing essential nutrients that are vital for human survival [4,5].

*Apis cerana*. Fabricius (*A. cerana*), the native honey bee of China, is widely distributed in the country [6,7]. In 2008, the number of *A. cerana* colonies in China was 2.8 million, with 5.16 million in 2014 [8]. By the end of 2016, the number of *A. cerana* colonies was close to 6 million [9]. This reflects the increasingly important role of *A. cerana* in modern agriculture [10]. Consequently, conservation and breeding of *A. cerana* has become more important. Due to different climatic conditions and geographical environments, the biological characteristics and management of *A. cerana* are different across various regions [11,12,13]. During the long-term natural selection process, *A. cerana* has developed strong adaptability to local environments and evolved excellent traits [14]. Based on the distribution range of *A. cerana*, it has been divided into 10 geographical subspecies in China, each with its own characteristics [7]. For example, the body size of the Aba Chinese bee is large, and it adapts to the climate and the nectar sources in plateau environments with altitudes of more than 2000 m [15]. The Southern Chinese bee performs well in hot climate environments [16]. The body size of the Hainan island Chinese bee is relatively small, and it has adapted to the climate and the nectar sources environment of the island [17]. In general, *A. cerana* bees are good at collecting scattered nectar sources and have strong resistance to *Varroa destructor* [18]. They also adapt well to low temperatures [19]. However, in *A. cerana*, the honey yield is lower, and the colony size is smaller than *Apis mellifera ligustica* (*A. m. ligustica*) [20]. This affects beekeepers’ willingness to manage *A. cerana*. Therefore, improvement of trait performance of *A. cerana* is the most important breeding goal. Currently, neither quantitative genetic breeding nor molecular genetic breeding is practiced in *A. cerana* in China. Instead, beekeepers’ selecting of queens is only based on their beekeeping experience. However, in this way, genetic improvement is nearly impossible to achieve. Therefore, it is necessary to investigate the current basic beekeeping management of *A. cerana* to facilitate the development of breeding.

In this study, we investigated the beekeeping management of *A. cerana* in eastern, southern, northwestern and central China. The importance of traits, biological characteristics, environment and colony management were analyzed. Further, the difference in management between *A. cerana* and *A. m. ligustica* in the same region were compared. This study provides beekeeping information of *A. cerana* in China, which is helpful to optimize breeding program success.

## 2. Materials and Methods

### 2.1. Survey Area

**Figure 1:**
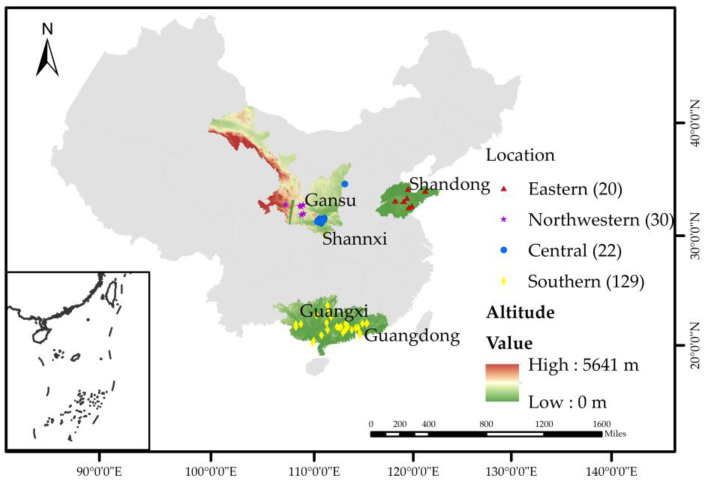
The surveyed area of *A. cerana*.

**Figure 2:**
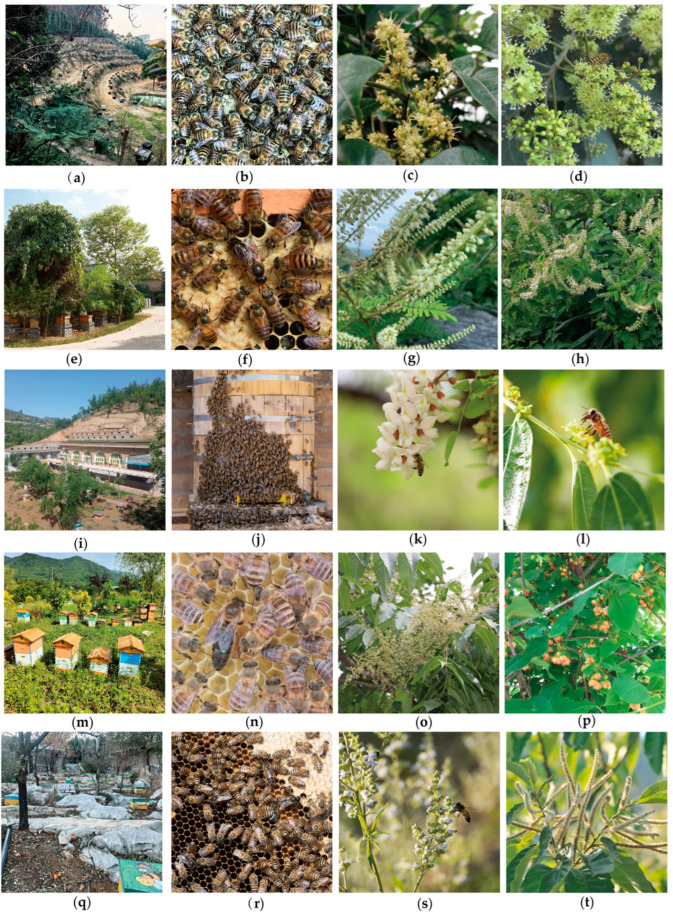
Classical apiaries, colonies and nectar plants in the surveyed regions. (**a**) The apiary of *A. cerana* in Guangdong. (**b**) The colony of *A. cerana* in Guangdong. (**c**) *Dimocarpus longan* Lour in Guangdong. (**d**) *Heptapleurum heptaphyllum* (L.) Y. F. Deng in Guangdong. (**e**) The apiary of *A. cerana* apiary in Guangxi. (**f**) The colony of *A. cerana* in Guangxi. (**g**) *Pterolobium punctatum* Hemsl in Guangxi. (**h**) *Phanera championii* Benth in Guangxi. (**i**) The apiary of *A. cerana* apiary in Shaanxi. (**j**) The colony of *A. cerana* in Shaanxi. (**k**) *Robinia pseudoacacia* L in Shaanxi. (**l**) *Ziziphus jujuba* Mill in Shaanxi. (**m**) The apiary of *A. cerana* in Gansu. (**n**) The colony of *A. cerana* in Gansu. (**o**) *Rhus chinensis* Mill in Gansu. (**p**) *Schisandra chinensis* (Turcz.) Baill in Gansu. (**q**) The apiary of *A. cerana* in Shandong. (**r**) The colony of *A. cerana* in Shandong. (**s**) *Vitex negundo var. heterophylla* (Franch.) Rehd in Shandong. (**t**) *Castanea mollissima* Blume in Shandong.

The surveyed regions included southern China (Guangdong province and Guangxi Zhuang autonomous region), central China (Shaanxi province), northwestern China (Gansu province) and eastern China (Shandong province) (Figure 1). In the surveyed regions, the southern China has a subtropical monsoon climate, central China features a north subtropical semi-humid climate, northwestern China experiences semi-humid and semi-arid monsoon climates and eastern China is characterized by a temperate monsoon climate. Apiaries, colonies and nectar plants in surveyed regions are shown in Figure 2.

### 2.2. Survey and Data Acquisition

**Figure 3:**
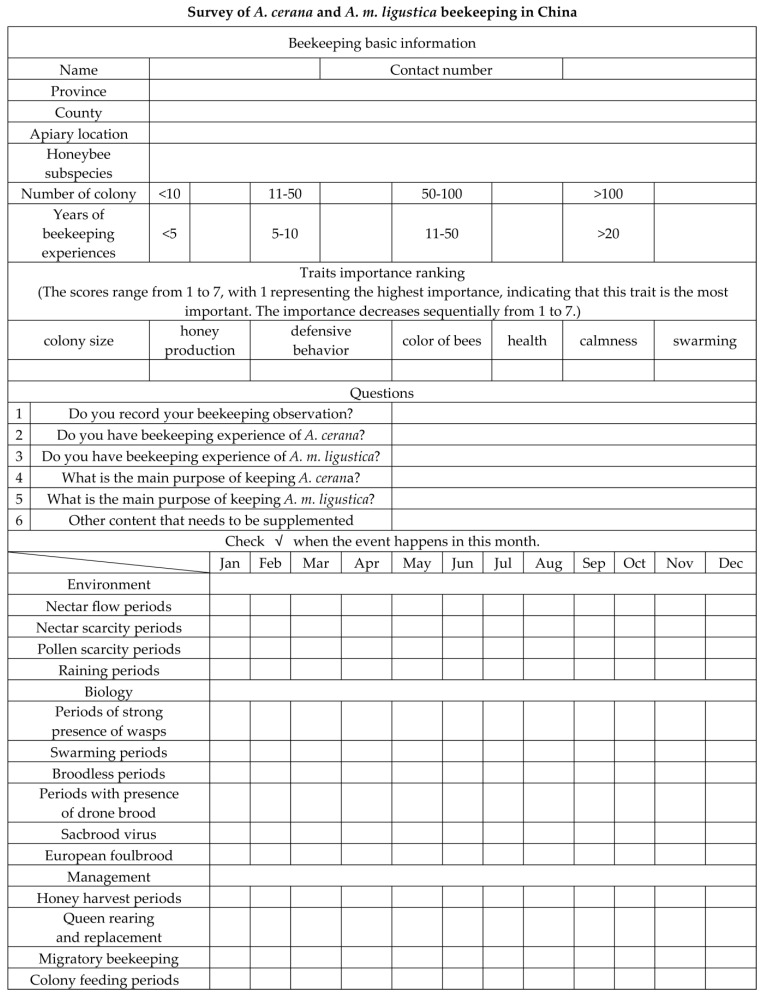
An example of a questionnaire designed for *A. cerana* and *A. m. ligustica* beekeeping in China.

The questionnaire of our survey was designed to obtain basic information on *A. cerana* apicultural management in China. The questionnaire was divided into three parts, including basic questions for apiary information, biological characteristics and management of colonies. All the data from this survey were collected through the questionnaire, as shown below (Figure 3). Paper versions of the questionnaire were distributed to local beekeeping organizations, which delegated surveyors to randomly select and communicate with beekeepers face-to-face in daily visits or at an annual conference. All the completed questionnaires were then returned to our center for data reduction and analysis.

For statistical analyses, the Microsoft^®^ Excel^®^ 2021 MSO software was used. Basic statistical tools were utilized to describe the data, such as frequencies or mean values. The rank frequency of each trait was counted to show the trait importance. The rank ranged from 1 to 7, with 1 representing the highest importance, indicating that this trait is the most important. Then, the traits’ average scores were calculated. Being ranked first in importance is assigned scores of 7, and being ranked last is assigned scores of 1. The importance scores were obtained based on comprehensive scores. To visualize the management measures duration results of colonies, the color level was carried out from light to dark based on the minimum to maximum frequency of the management measures occurring in the corresponding month.

Spearman correlations were determined with OriginPro^®^ 2024b in order to assess the relations between the management measures of colonies. This coefficient varies from −1 to 1. The strength of the correlations was evaluated according to the following limits: 0.1 < |r| < 0.3: low correlation; 0.3 < |r| < 0.5: medium correlation; |r| > 0.5: high correlation. ** *p*-value ≤ 0.01: correlation is significant at the 0.01 level. * *p*-value ≤ 0.05: correlation is significant at the 0.05 level.

## 3. Results

### 3.1. Apiary Basic Information of A. cerana

**Figure 4:**
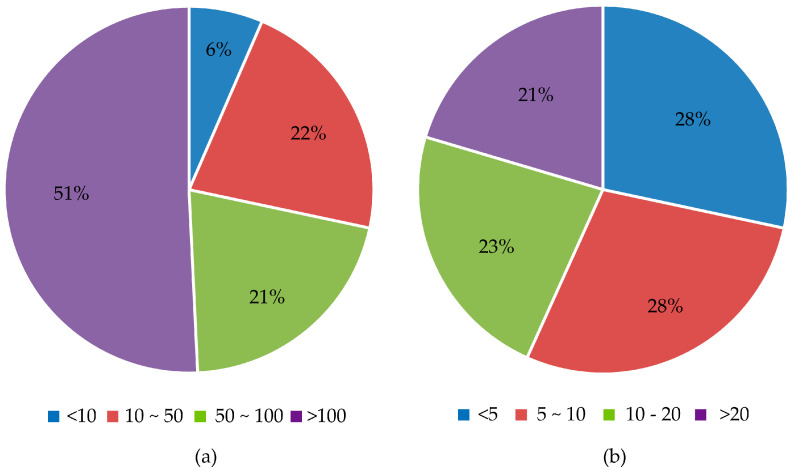
Analysis of the apiaries’ scale and the beekeepers’ beekeeping experience. (**a**) The scale of the apiaries; (**b**) beekeepers’ experience in beekeeping.

There were 201 apiaries breeding *A. cerana* participating in the survey. The total number of colonies was 24,890 (Figure 4). Apiaries with more than 100 colonies accounted for 50.7%. As a comparison, there were 50 apiaries breeding *A. m. ligustica* participating in the survey, with a total of 1100 colonies. For the experience of *A. cerana* beekeepers, 28% of beekeepers had less than 5 years of experience, 28.4% of beekeepers had 5 to 10 years of beekeeping experience and 22.9% of beekeepers had 10 to 20 years’ experience. Beekeepers with more than 20 years of beekeeping experience account for 20% of the total. Beekeepers using notebooks for recording accounted for 48% of all beekeepers surveyed. Almost half of the beekeepers keep records.

### 3.2. Trait Importance Ranking of A. cerana

**Figure 5:**
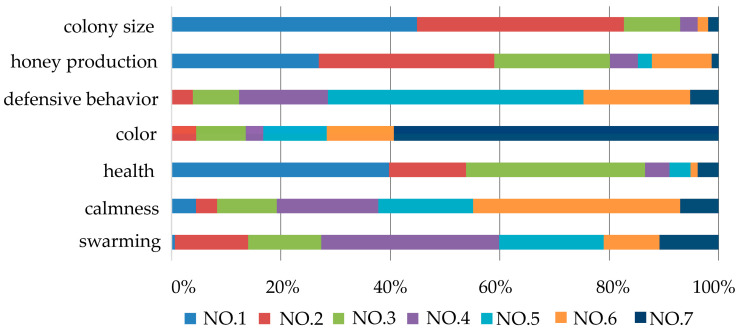
Traits’ importance ranking of *A. cerana*. NO.1, ranked first in importance; NO.2, ranked second in importance; NO.3, ranked third in importance; NO.4, ranked fourth in importance; NO.5, ranked fifth in importance; NO.6, ranked sixth in importance; NO.7, ranked seventh in importance.

The important traits of *A. cerana* for beekeepers was investigated (Figure 5 and Table 1). The results showed that the top three traits are colony size, colony health and honey production (Figure 5 and Table 1). There are slight differences among different regions. In northwestern China, beekeepers prioritize colony health, followed by honey production and thirdly, colony size. The traits ranked fourth to sixth are swarming, calmness and defensive behavior. The color of bees ranked the last. Colony health is the basis of the colony development, and a large colony could produce more honey. Honey is the main product of *A. cerana* in China. In large-scale apiaries, colonies are another important product. The results indicated that the main purpose of beekeepers keeping *A. cerana* is to obtain economic benefits.

### 3.3. Environments of A. cerana

**Figure 6:**
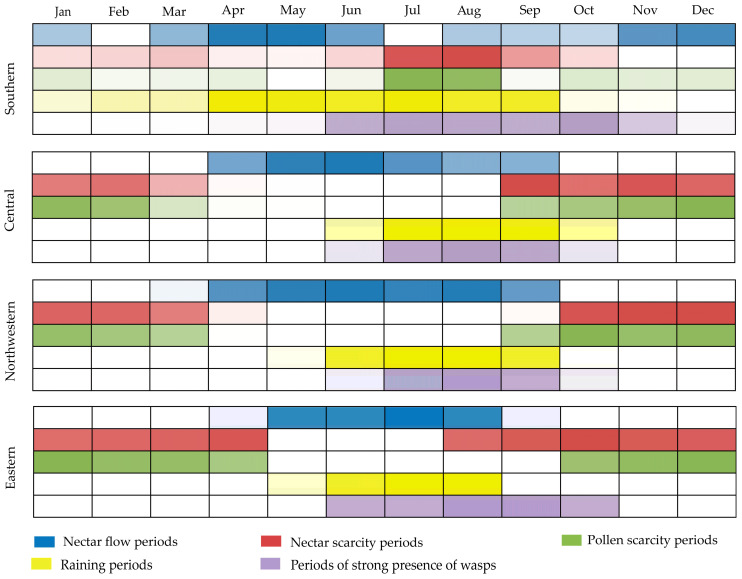
The living environments of *A. cerana* in China.

In southern China, there are two nectar flow periods. One is from March to June, and the other is from late August to January of the following year (Figure 6). In other regions, the nectar flow lasts from April to September (Figure 6). Despite the tropical and subtropical climates of southern regions, which support flowering plants throughout the year, honey bees still face periods of pollen and nectar scarcity, primarily occurring in July to August and March. For the eastern, central and northwestern regions, these scarcity periods occur during autumn, winter and spring. Across all regions, there is a partial overlap between the rainy season and the nectar flow period. Prolonged rainy seasons hinder bees’ foraging activities. Additionally, during the high temperatures of summer, wasps pose a threat to *A. cerana*.

### 3.4. Biology of A. cerana

**Figure 7:**
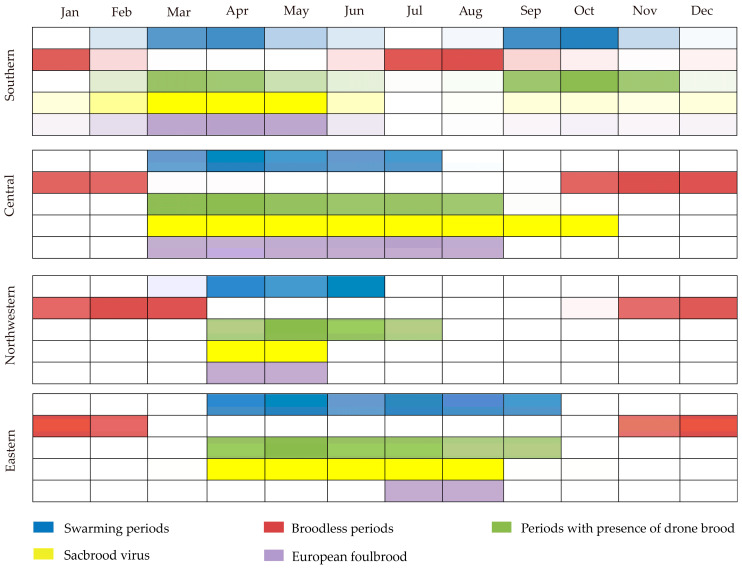
The biology of *A. cerana* in China.

In southern regions, coinciding with the two nectar flows, there are two swarming periods (Figure 7). One is from March to April, and the other is from September to November (Figure 7). In other regions, there is only one swarming period, concentrated in the spring (Figure 7). The period with the drone brood is created, overlapping with the one of swarming. This ensures that there are enough mature drones for virgins to mate with. Broodless periods overlap with periods of nectar and pollen scarcity. Sacbrood virus and European foulbrood mostly occur during the spring breeding stage, but only a few apiaries reported Sacbrood virus and European foulbrood.

### 3.5. Management of A. cerana

**Figure 8:**
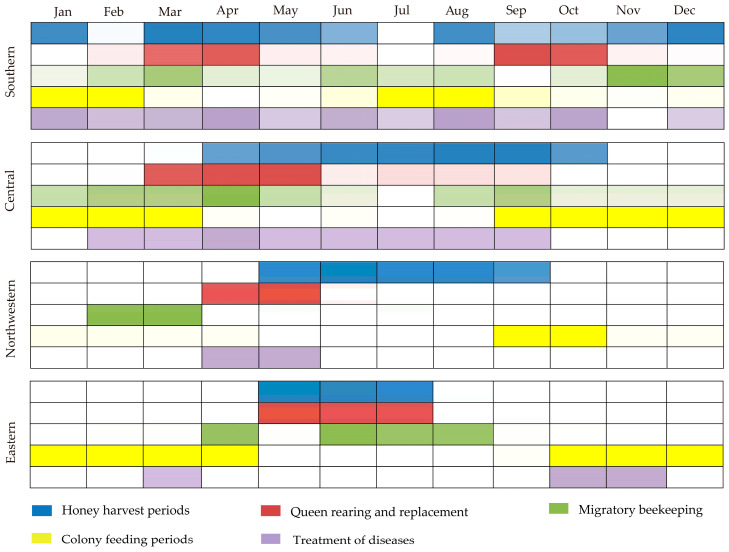
The management of *A. cerana* in China.

The honey harvesting period typically follows the nectar flow period. In southern regions, there are two peak honey harvesting seasons: one from March to June, and the other from August to January of the next year (Figure 8). In other areas, the honey harvesting season lasts from April to October (Figure 8). The timing for queen rearing, replacement and mating coincides generally with the period of drone brood production and swarming (Figure 8). To ensure optimal honey production, beekeepers should conduct queen rearing and replacement prior to the main nectar flow, allowing the colony to develop strongly. Migratory beekeeping occurs throughout the year, indicating that the migratory beekeeping of *A. cerana* has become common in China. Feeding usually takes place during periods of pollen scarcity. The treatment of diseases overlaps with the reproductive period and is related to the incidence of larval stage diseases. Consequently, as few diseases were reported, there have been correspondingly few apiaries reporting any treatment.

### 3.6. Spearman Correlation Analysis of Apiculture of A. cerana

**Figure 9:**
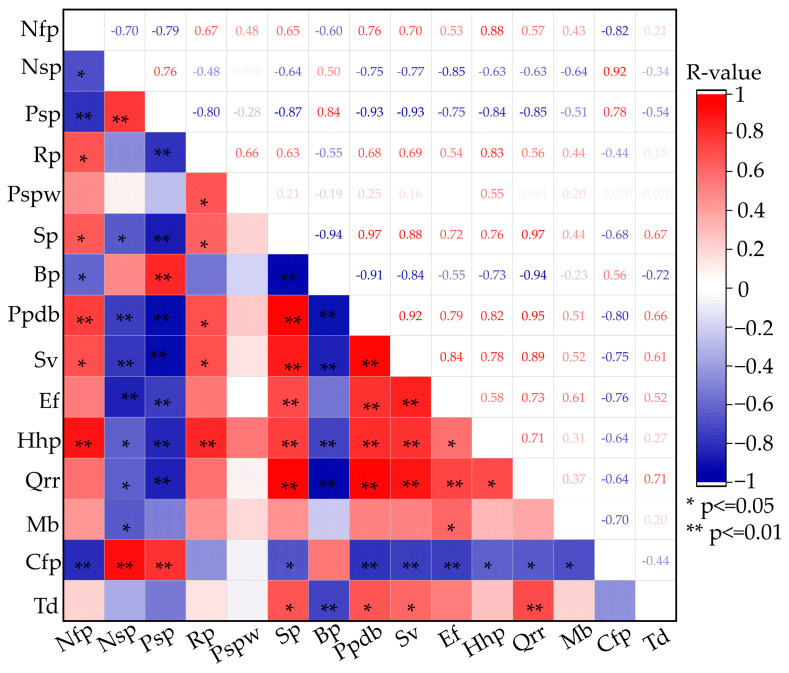
Heatmap of Spearman’s correlation of apiculture of *A. cerana*. R-value varies from −1 to 1. Blue, negative correlation; red, positive correlation. The darker the color, the stronger the correlation. ** *p*-value ≤ 0.01, correlation is significant at the 0.01 level. * *p*-value ≤ 0.05, correlation is significant at the 0.05 level. Nfp, nectar flow periods; Nsp, nectar scarcity periods; Psp, pollen scarcity periods; Rp, raining periods; Pspw, periods of strong presence of wasps; Sp, swarming periods; Bp, broodless periods; Ppdb, periods with presence of drone brood; Sv, Sacbrood virus; Ef, European foulbrood; Hhp, honey harvest periods; Qrr, queen rearing and replacement; Mb, migratory beekeeping; Cfp, colony feeding periods; Td, treatment of diseases.

The effect of environments on biology and the management of *A. cerana* is evident. The correlation analysis between environment, biology and management can help beekeepers better manage *A. cerana* colonies. The results showed that swarming periods and the periods with the presence of the drone brood are highly positively correlated with queen rearing and replacement (Figure 9). It indicated that the beekeepers mastered the best time to produce queens. The presence of Sacbrood virus and European foulbrood are highly positively correlated with the periods with the presence of the drone brood. It indicated that the brood diseases could be reduced by controlling the presence of the drone brood.

### 3.7. Apiculture of A. cerana vs A. m. ligustica

**Figure 10:**
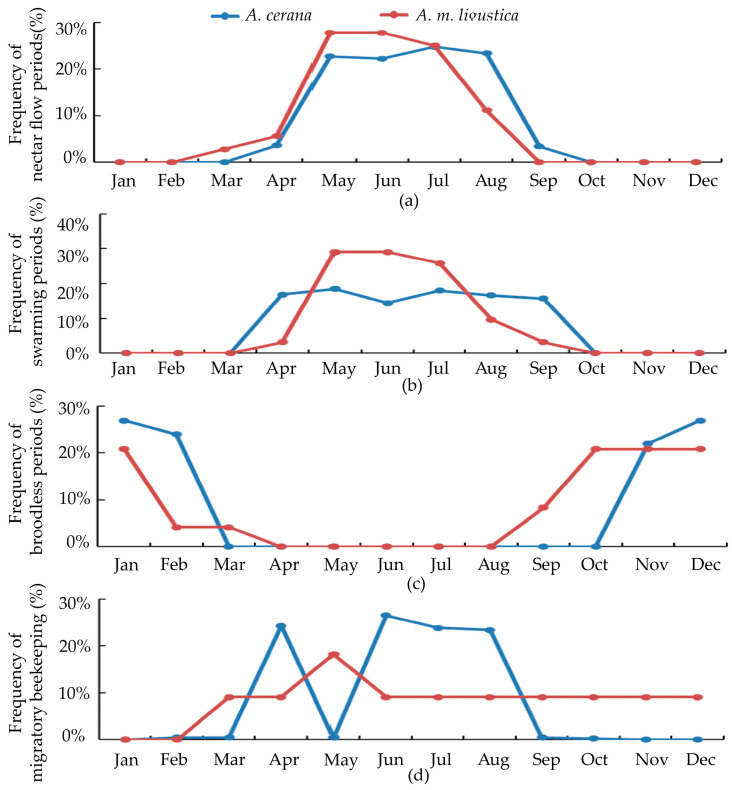
The apiculture difference between *A. m. ligustica* and *A. cerana* in eastern China. (**a**) Nectar flow periods; (**b**) swarming periods; (**c**) broodless periods; (**d**) migratory beekeeping.

The surveyed regions in southern and central China predominantly keep *A. cerana*. In contrast, the surveyed regions in northwestern and eastern China not only keep *A. cerana* but also host a significant number of beekeepers who keep *A. m. ligustica*. Consequently, we also conducted an investigation into the apiculture of *A. m. ligustica* and compared the data obtained between *A. cerana* and *A. m. ligustica* (Figure 10 and Figure S1).

The results are almost the same between the important traits of *A. m. ligustica* and *A. cerana.* The top three are colony size, honey production and health. For the environmental factors, it is also the same between *A. m. ligustica* and *A. cerana*. For *A. cerana*, the period with nectar is longer, because *A. cerana* are better at utilizing scattered nectar and more active at low temperatures. For biological characteristics, *A. cerana* queens stopped laying eggs before winter at later dates than *A. m. ligustica*. This also indicates that *A. cerana* is better suited to lower temperatures. The swarming period is longer for *A. cerana,* which leads to smaller colony sizes compared to *A. m. ligustica*. The management of *A. cerana* and *A. m. ligustica* is closely related to their biological characteristics. Migratory beekeeping is more common in *A. m. ligustica*.

## 4. Discussion

*Apis cerana* is the native honey bee in China and is important to ecological balance [21]. It is gratifying that the number of colonies of *A. cerana* has been increasing in China over the past 15 years [22]. This reflects the increasingly important role of *A. cerana* in China’s modern agriculture [14]. In this study, we surveyed traits, the biological environment and management of *A. cerana* in different regions of China. The results should help to optimize the breeding programs of *A. cerana* and further contribute to substantive breeding accomplishments with honey bees which have enhanced their role in modern agriculture.

Data are the foundation for breeding programs. In this study, we found that more than 50% of the surveyed apiaries contain over 100 colonies. Almost half of the beekeepers keep records using notebooks. This indicates that the breeding business for *A. cerana* is gradually developing towards a large scale, and the beekeepers are paying more attention to data. This is beneficial for the future development of the breeding program of *A. cerana*.

A successful honey bee breeding program should first examine the specific needs of the particular region in which the stock they develop is used. Only in this way can breeding programs be initiated with useful goals. This examination includes identifying the expected sources of receipts from beekeeping, the adverse beekeeping conditions in the area and the apparent shortcomings of existing stocks of bees. Well-founded breeding programs have emphasized goals appropriate to the economic needs of specific regions. Our results showed that the important top three traits identified by beekeepers are colony size, colony health and honey production. This corresponds to the fact that income in beekeeping in these regions arises from the sale of honey and package bees. Consequently, colony size, honey production and health would be the desirable goal of breeding programs.

In addition to the potential sources of income, the biological environment of a beekeeping area often has been important to breeding programs. Continuous nectar flow [23], absence of bee diseases [24] and parasites [25,26] are valued factors in a successful honey bee industry. In the central, eastern and northwestern regions surveyed, it was found that nectar flows are mainly observed in spring and summer. In the southern areas, there are two nectar flows annually, with an additional one in winter. In the southern regions, queen rearing and replacement also occur twice. Therefore, carrying out breeding programs in the southern region would achieve two generations each year, consequently accelerating genetic improvement. Furthermore, there are few reports of honey bee diseases and parasites in all surveyed regions. This is a valuable attribute factor for breeding programs.

The biological characteristics of *A. cerana* should be utilized by breeders to breed specific lines. Our results showed that for *A. cerana,* the nectar flow period is longer than that in *A. m. ligustica*. As stated previously, this indicates that *A. cerana* is good at discovering scattered nectar sources and is adapted to low temperatures. These findings are consistent with previous studies [27,28,29]. These biological characteristics are crucial for the pollination of plants that bloom in winter or grow in unique ecological environments. Breeding for these characteristics in *A. cerana*, breeders could develop lines suited to low temperature or specific environments. Our results also showed that compared with *A. m. ligustica,* the swarming period of *A. cerana* lasts longer. This is consistent with previous research, which suggests that *A. cerana* have a stronger swarming ability than *A. m. ligustica*. A strong swarming ability tends to hinder colony size [30]. If the breeding goal is to improve bee production or large colony size, breeders should select colonies with low swarming ability [31,32].

Based on surveys of the biological environment, queen rearing could be carried out from spring to autumn in these areas. In spring, the natural swarming season, with warm climate and abundant honey source, the colonies have developed to sufficient swarm potential, a large number of young workers have accumulated in the hive and the drones have also begun to emerge in large numbers. This period is the best period for artificial breeding of queens. Rearing and replacing queens in spring is better because there are more drones, resulting in more frequent and better-quality mating [33]. At this time, larval acceptance rate is also high. Using new queens that have just laid eggs in new colonies will result in strong colonies that can quickly be put into production. Therefore, we suggest that the artificial queen rearing time should be determined according to the local climate and honey source conditions under the production and breeding requirements, so that new colonies can develop into strong colonies before the arrival of the nectar flow period. In addition, in order to mate with the queens, colonies should be selected to rear drones, so that there are enough drones during the mating period.

Our survey of beekeeping in *A. cerana* and *A. m. ligustica* in the same regions showed that there is competition for nectar and colony reproduction between them. The competition from *A. m. ligustica* affects the population size of *A. cerana* [34]. *Apis cerana* and *A. m. ligustica* are closely related species with overlapping ecological niches, resulting in intense interspecific competition [35]. Our investigation on *A. cerana* and *A. m. ligustica* in the same region revealed that their nectar and pollen flow overlap. Nectar and pollen are the basis for the survival of bees [36,37]. Previous research has demonstrated that individual *A. m. ligustica* bees had greater resource-holding potential than *A. cerana*, and the temporal overlap of them at flowers could result in *A. m. ligustica* becoming the dominant forager [38,39]. In the competition for nectar and pollen, *A. cerana* is at a disadvantage. It is difficult for *A. cerana* to survive in regions where *A. m. ligustica* is intensively managed. The intensive foraging of *A. m. ligustica* leads to its dominance of the major nectar flow. Although the colonies of *A. m. ligustica* moved away at the end of the nectar period, the local wild *A. cerana* colonies still face survival difficulties due to insufficient nectar and pollen stored during the main nectar period. Competition from *A. m. ligustica* apiaries reduces the honey production of *A. cerana*, leading to reduced numbers of beekeepers using *A. cerana*. In addition, the queen pheromones between *A. cerana* and *A. m. ligustica* are similar [40]. Our results showed that the periods of queen rearing and replacement also overlap between them. *Apis cerana* queens are often interfered with by *A. m. ligustica* drones during mating flights. In regions where *A. m. ligustica* populations are dominant, it is difficult for *A. cerana* queens to successfully mate. How to coordinate the reproduction of *A. cerana* and *A. m. ligustica* in the same region requires further research.

## 5. Conclusions

The worldwide value of the honey bee as an agricultural animal is increasingly being recognized. In this study, we investigated the important traits, biological environment and management of *A. cerana* in eastern, southern, northwestern and central China. The results showed that the most favored traits for beekeepers are colony size, colony health and honey production. Compared with *A. m. ligustica*, *A. cerana* is better adapted to low temperatures and scattered nectar sources. Our results should help to optimize the breeding programs of *A. cerana* and further contribute to substantive breeding accomplishments with honey bees which have enhanced their role in modern agriculture.

## Figures and Tables

**Table 1:** Traits’ average scores of different regions and importance ranking of *A. cerana* based on comprehensive scores.

Traits	Southern	Central	Northwestern	Eastern	Comprehensive Scores	Rank
colony size	6.00	6.28	5.00	6.25	5.96	1
health	5.97	5.22	6.53	6.10	5.87	2
honey production	5.68	5.15	6.50	4.20	5.54	3
swarming	3.54	3.52	3.97	3.70	3.61	4
defensive behavior	2.78	3.20	1.30	3.95	2.78	5
calmness	2.70	2.70	3.03	2.55	2.73	6
color	1.34	1.93	1.73	1.25	1.53	7

## Data Availability

No new data were created or analyzed in this study. Data sharing is not applicable to this article.

## Supplementary Materials

The following supporting information can be downloaded at: https://www.mdpi.com/article/10.3390/life15010009/s1, Figure S1: The apiculture difference between *A. m. ligustica* and *A. cerana* in northwestern China.
