# Supplementary material for: Investigations on Beekeeping and Breeding of Apis cerana in China
Source: Life (Basel). 2024 Dec 25;15(1):9. doi: 10.3390/life15010009 (PMC11767041; doi:10.3390/life15010009)
Supplement: Supplementary file 1 [file life-15-00009-s001.zip › life-3362043-supplementary.pdf]

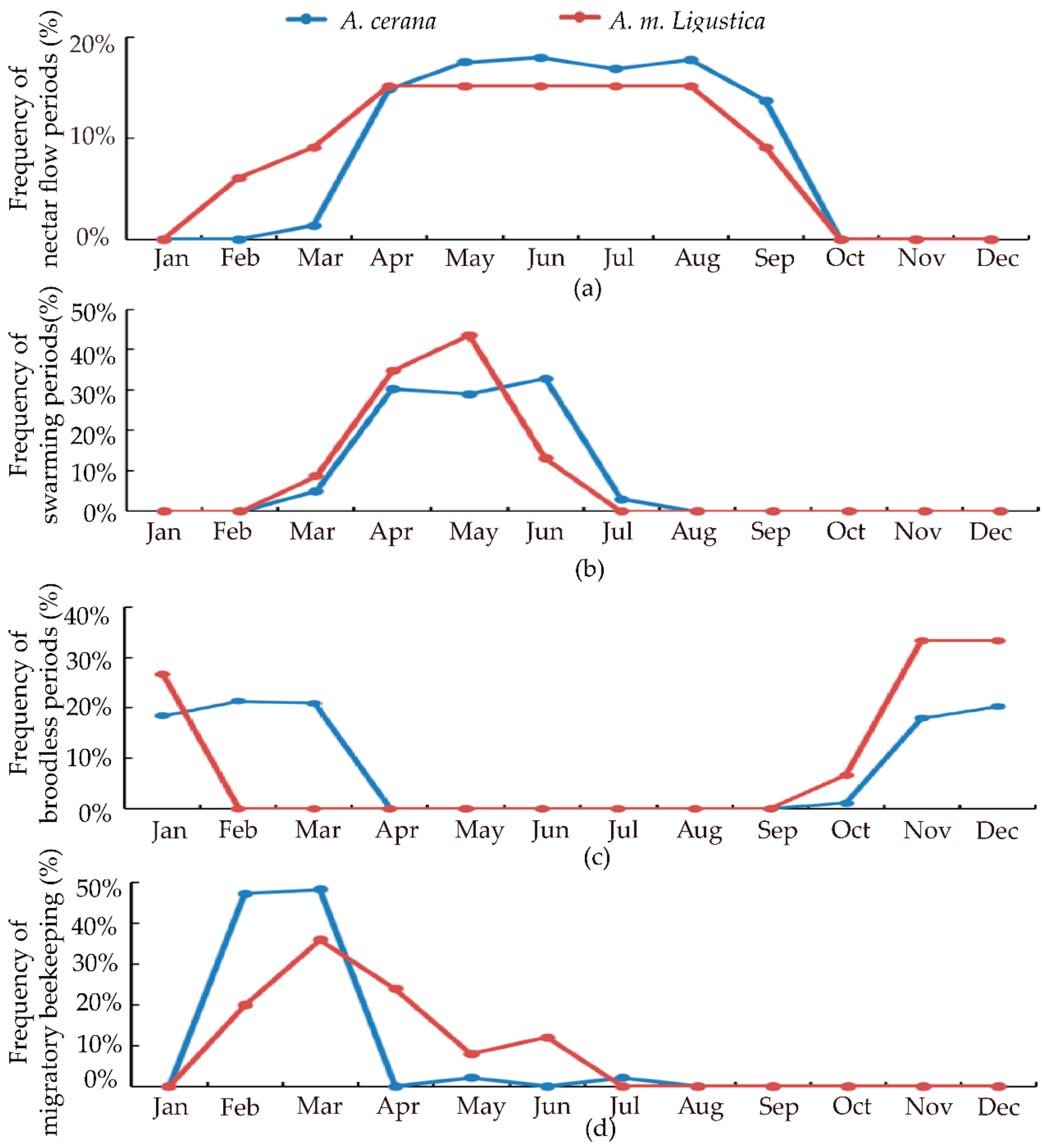

Figure S1. The apiculture difference between *A. m. ligustica* and *A. cerana* in northwestern China. (**a**) Nectar flow periods; (**b**) Swarming periods; (c) Broodless periods; (d) Migratory beekeeping.
